# J. Y. on Mr. Brown's Observations on "Zoonomia"

**Published:** 1799-10

**Authors:** 


### *To the Editors of the Medical and Phyfical Journal.*

GENTLEMEN,

I TAKE the liberty, through the medium of your valuable *Journal*, to correct a mifftatement of Dr. DARWIN's principles, made by Mr. BROWN, the ingenious author of "*Obfervations on 'Zoonomia,*" fome time ago publifhed in in Edinburgh. This miſtatement occurs in the firſt chapter of that work, in which the author endeavours to prove, that according to Dr. Darwin's principles, the original production of ſenſorial power is impoſſible.

Dr. Darwin, ſuppoſes, "that ſenſorial power, or the ſpirit of animation, is the immediate cauſe of the contraction of animal fibres, and is liable to general or partial diminution, or accumulation; that the quantity expended in the continual motions of life, is ſupplied by the ſecretion or production of it in the brain and ſpinal marrow, while, at the ſame time, a certain quantity of ſenſorial power is neceſſary for the action of a hand." From theſe circumſtances, Mr. Brown thinks that he can prove the fallacy of Dr. Darwin's reaſoning. "For, (ſays he) in order to call ſenſorial power into exiſtence, it is neceſſary that it previouſly exiſt in the brain and ſpinal marrow, as much as in the glands which ſecrete any other fluid. The thing ſecreted muſt therefore exiſt before the organ which ſecretes it can be called into action."

Mr. Brown is aware of an objection which may be oppoſed to his reaſoning, viz. that the embryo may derive a ſmall portion of ſenſorial power from the parent, and thus be capable of increaſing the quantity by ſecretion. But this objection he thinks cannot be admitted, " becauſe (ſays he) the embryo, according to Darwin, is a ſimple filament, without ſenſorial power, or the means of producing it."

In this, however, conſiſts Mr. Brown's error; for on examining the chapter on Generation of the '*Zoonomia*,' p. 480, he will find that Dr. Darwin thinks "that the embryo, at the earlieſt period of its exiſtence, as ſecreted from the blood of the male, would ſeem to conſiſt of a *living filament*, with certain capabilities of irritation, ſenſation, volition, and aſſociation," and this opinion he adopts, though he thinks it " difficult to be conceived how a living entity, which this embryo is, can be ſeparated or produced from the blood by the action of a gland."

Another quotation from the '*Zoonomia*,' p. 492, will ſtill further prove Mr. Brown's miſconception of Dr. Darwin's idea. " I conceive the primordium or rudiment of the embryo (ſays Dr. Darwin), as ſecreted from the blood of the parent, to conſiſt of a *ſimple living filament*, as a muſcular fibre, &c. and I ſuppoſe this living filament, of whatever form it may be, to be endowed with the capability of being excited into action by certain kinds of ſtimuli."

*Auguſt 6th*, 1799. J. Y.

